# Supplementary material for: Photothermal Effect in Plasmonic Nanotip for LSPR Sensing
Source: Sensors (Basel). 2020 Jan 25;20(3):671. doi: 10.3390/s20030671 (PMC7039235; doi:10.3390/s20030671)
Supplement: Supplementary file 1 [file sensors-20-00671-s001.pdf]

sensors

Supplementary

Photothermal Effect in Plasmonic Nanotip for LSPR Sensing

Muhammad Shemyal Nisar [1,2], Siyu Kang [1,2] and Xiangwei Zhao [1,2,*]

1 State Key Laboratory of Bioelectronics, School of Biological Science and Medical Engineering, Southeast University, Nanjing 210096, China; mshemyalnisar@hust.edu.cn (M.S.N.); 220191928@seu.edu.cn (S.K.)
2 Southeast University-Shenzhen Research Institute, Shenzhen 518000, China
* Correspondence: xwzhao@seu.edu.cn

Received: 24 December 2019; Accepted: 21 January 2020; Published: 25 January 2020

Abstract: The influence of heat generation on the conventional process of LSPR based sensing has not been explored yet. Thus, a need exists to draw attention towards heat generation issue during LSPR sensing as it may affect the refractive index of analyte, leading to incorrect sensory conclusions. This manuscript addresses the connection between photo-thermal effect and LSPR. We numerically analyze the heat performance of a gold cladded nano-tip. The numerical results predict a change in the micro-scale temperature in the microenvironment near the nano-tip. These numerical results predict temperature increase of more than 20 K near the apex of the nanotip which depends on numerous factors including input optical power and the diameter of the tip. We analytically show that this change in the temperature influences a change in the refractive index of the microenvironment in the vicinity of the nano-tip. In accordance with our numerical and analytical findings, we experimentally show an LSPR shift induced by a change in the Input power of the source. We believe that our work will shed paradigm shifting light on the whole enterprise of LSPR based sensing.

Keywords: Optical Fiber Sensors; Localized Surface Plasmon Resonance; Nanotip; Electromagnetic Heating; Refractive Index

---

Description of Simulation Setup

We performed FEM based multiphysics simulations on commercially available software. We used a module specifically designed for our purposes called “Microwave Heating”. The Module allows us to use electromagnetic excitation source and then calculates the resulting rise in the temperature. The solution of the electromagnetic part is carried out in frequency domain while the that of heating part is carried out in the time domain. The time domain simulation was conducted on the time range of zero to 0.3 seconds with an incremental step of 0.01seconds. The simulation design was setup in accordance with similar work done by the Team of Naomi Halas[1], although with appropriate alterations to their design. Given we are simulating a tapered fiber tip, this makes our design spatially large. Therefore, in the interest of minimizing computational resources required, we used a 2D-Axisymeteric model where we modeled the fiber tip with its gold cladding, and the environment. The design can thus be divided into two parts; the design of the tapered fiber, and the design of the environment.

Designing the Environment:

Under the assumption that the stead state temperature remains the same irrespective of the medium, we used air as our environment for the purpose of simulations although the results were randomly corroborated with other chemicals such as methanol, ethanol, water and IPA. Temperature

boundary was applied to the boundaries of the environment as in the far field the temperature remains the constant at the room temperature. The room temperature was chosen to be 293.15K. A buffer region of a few 2 microns was added at the point where the tip crosses over the temperature boundary. This was done due to computational requirements of the software as otherwise the temperature boundary would effectively apply temperature on the gold layer as well.

Designing the Gold-Cladded Tapered Fiber Tip:

The design for a tapered tip was made similar to the already published work[2] as well. We made the same design in a 2D-axisymmeteric model. Silica was used as the material of the fiber core, and the cladding was a 50nm thick layer of gold. The tapper was created such that the tip was 300nm in diameter, the fiber that we use is a multimode fiber with a diameter of 40 microns. The length of the taper was 55.5 microns. The design is given in Figure S1a an image of the meshed structure is shown in the Figure S2b. The structure was simulated with physics controlled extremely fine mesh so that we can accuracy of the results is not compromised and at the same time get results from the limited computational resources at hand. The input power was chosen to reflect the actual power in the experiment with TEM polarization, while boundary at the far end was chosen to be "Perfect Electric Conductor" for electromagnetic simulation. For the thermal calculations, "Temperature" boundary was used with the temperature of 293.15K while the environment surrounding the gold cladded tip was set as a "Fluid" at 1 atm pressure. The parameters for gold and silica layer used in our model are given in the Table S1 given below, while that of the fiber design are given in Table S2.

Table S1. Details of material parameters of Gold and Silica used.

Parameters	Gold	Silica
Electrical Conductivity	45.6E6 S/m	1E-4 S/m
Heat Capacity, constant pressure	129 J/kg.K	703 J/kg.K
Density	19300 kg/m^3	2203 kg/m^3
Thermal Conductivity	317 W/m.K	1.38 W/m.K
Relative Permitivity	6.9	2.09
Relative Permeability	-0.19	1
Constant of Thermal Expansion	14.2E-6 K^{-1}	0.55E-6 K^{-1}

Table S2. Details of Simulation Setup.

Parameters	Value
Boundary Condition	Perfect Electric Conductor
Input Polarization	TEM

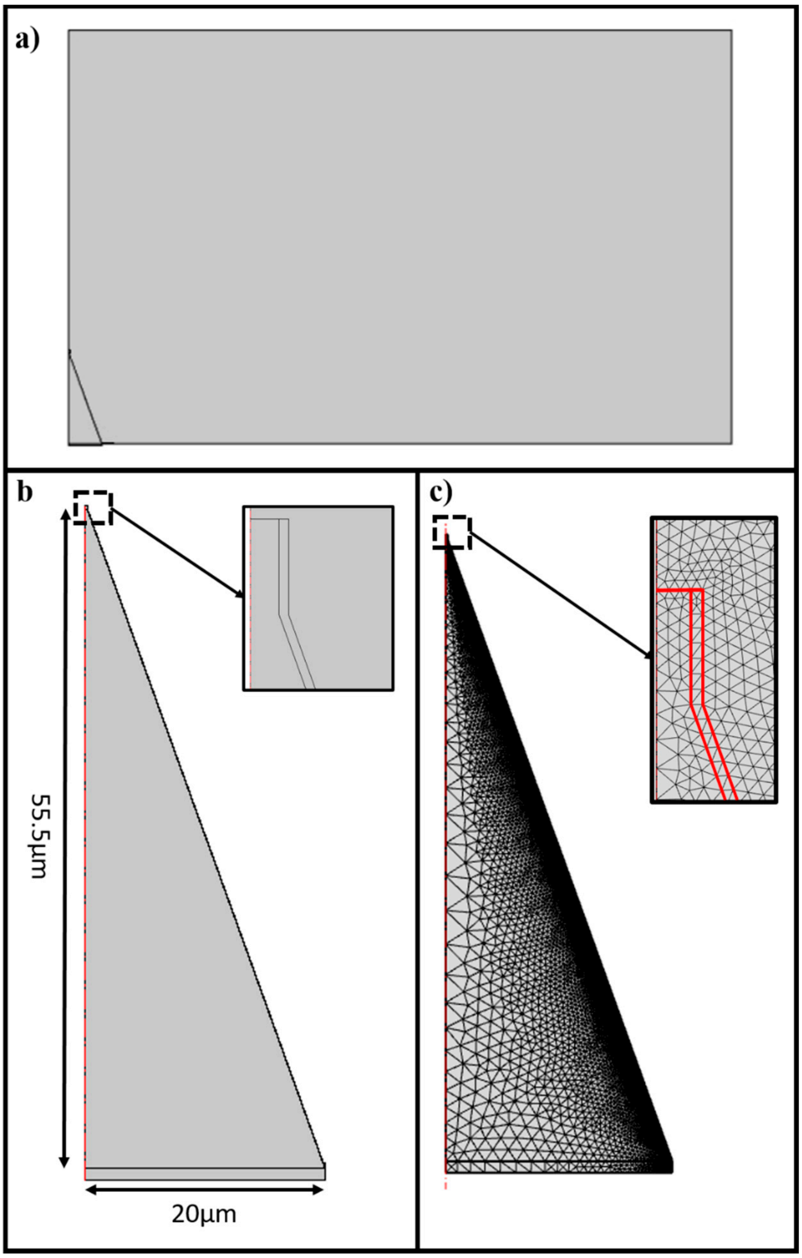

Figure S1. The complete schematic of the model used for simulation is given in figure a, with the fiber tip at the bottom left corner while the rest of the region is the environment. (The environment has been removed from the image for the sake of simplicity) b) The schematic of the fiber tip used for simulation the inset shows the enlarged image of the tip. c) The mesh on the fiber tip is shown while the inset is the enlarged image of the tip. The outline of the actual tip is highlighted in the image so that to make it discernable in the image. .

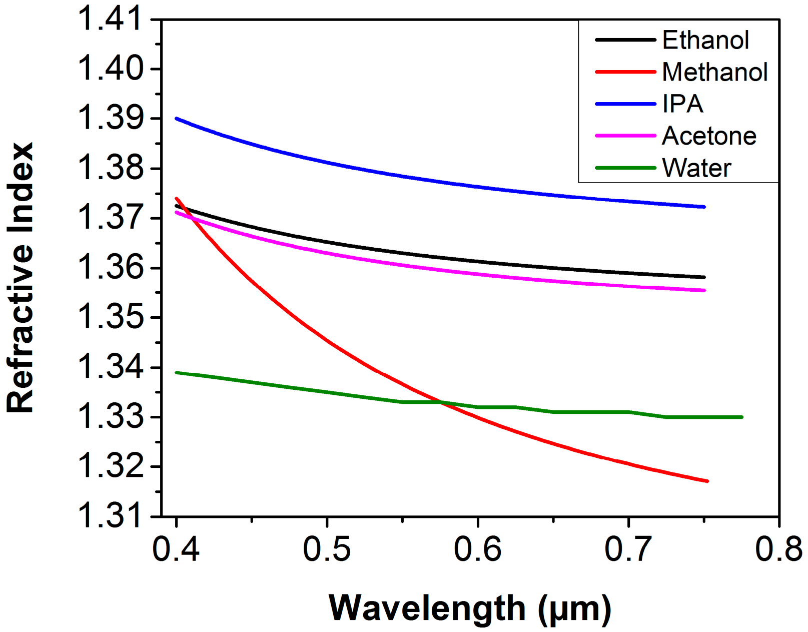

Figure S2. Relationship between refractive index and incident wavelength for our chemicals of interest.

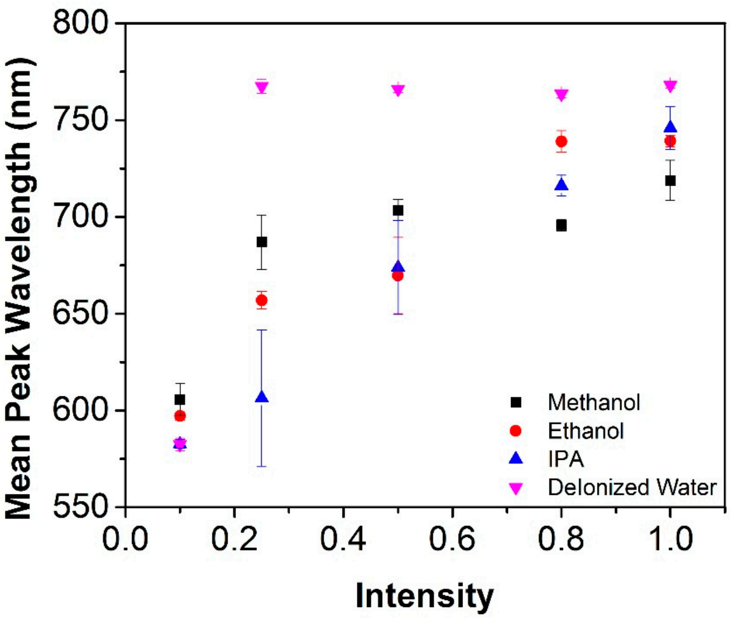

Figure S3. Scatterplot of Wavelength of LSPR Peak with respect to normalized intensity for each of the analyte chemicals including data points for 10% of the input light.

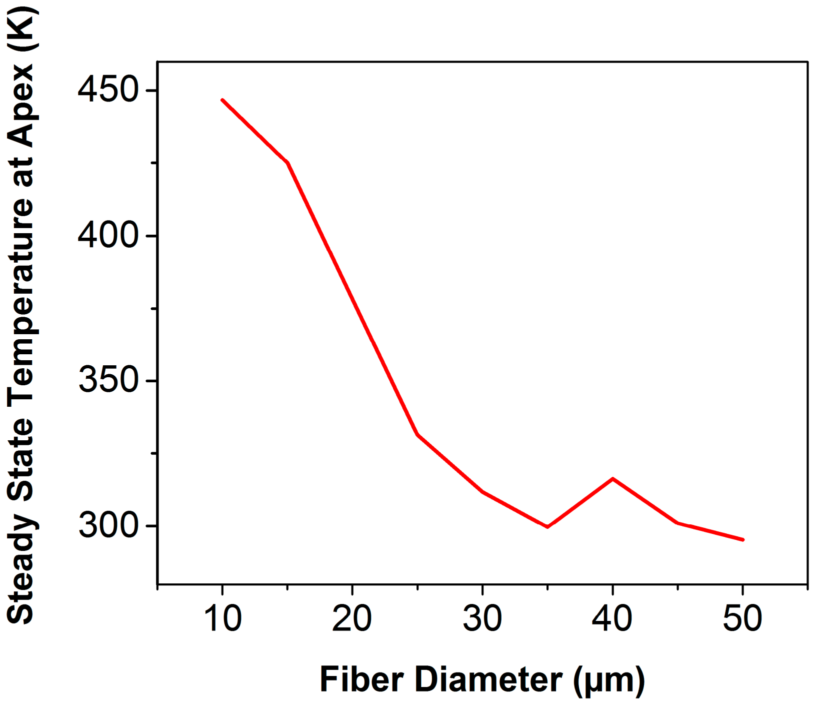

Figure S4. The graph shows the change in temperature with respect to the change in fiber diameter.

Table S3. Details of studies that the use LSPR giving the laser powers used in each.

The Source	Incident Power	Ref. # in Main Text
L7868-01, Hamamatsu, Japan	0.2 mW	[13]
Halogen Lamp of Olympus BX50	100 W	[14]
LS-1, Ocean Optics	6.5 W	[15,34]
Topica DI pro	105 mW	[19]
Nd:YAG laser	0.1-40 MW	[29]
He Ne Laser	4.1 W/cm^2	[36]
HL-2000, Ocean Optics	1 mW	[32,37,This study]

Table S4. Details of studies using Photothermal Effect giving the laser powers used in each.

Purpose	Incident Power	Ref. # in Main Text
Catalysis	250 mW/cm^2<br>40 kW, 50 mW	[44,45,46]
Cancer Therapy	100 mW, 10 mW, 2 mW<br>2 W/cm^2, 1 W/cm^2, 1.54 W/cm^2	[47,48,56,57,58,59]
Liquid Heating	2 mW, 5 mW-20 mW	[53,54]
Thermal Imaging	20 mW	[61]
Thermal LSPR	1µW/µm^2	[47]

References

1. Goodman, A.M.; Hogan, N.J.; Gottheim, S.; Li, C.; Clare, S.E.; Halas, N.J. Understanding Resonant Light-Triggered DNA Release from Plasmonic Nanoparticles. *ACS Nano* **2017**, *11*, 171–179.
2. Morozov, Y.M.; Lapchuk, A.S.; Gorbov, I. V.; Yao, S.-L.; Le, Z.-C. Optical plasmon nanostrip probe as an effective ultrashort pulse delivery system. *Opt. Express* **2019**, *27*, 13031–13052.
